# A new species of *Agelas* from the Zanzibar Archipelago, western Indian Ocean (Porifera, Demospongiae)

**DOI:** 10.3897/zookeys.553.5999

**Published:** 2016-01-14

**Authors:** Renata Manconi, Roberto Pronzato, Erica Perino

**Affiliations:** 1 Dipartimento di Scienze della Natura e del Territorio (Dip.Ne.T.), Università di Sassari, Via Muroni 25, I–07100, Sassari, Italy; 2 Dipartimento di Scienze della Terra dell’Ambiente e della Vita (Di.S.T.A.V.), Corso Europa 26, I–16132 Genova, Italy

**Keywords:** Biodiversity, sponges, morpho-taxonomy, diagnostic key, geographic range, Unguja Island

## Abstract

A new sponge species (Demospongiae: Agelasida: Agelasidae) is described from the eastern coast of Unguja Island in the Zanzibar Archipelago. *Agelas sansibarica* **sp. n**. is compared to all other *Agelas* species described so far. The new species differs from its congeners mainly in its three categories of verticillate spicules (acanthostyles, acanthostrongyles, and acanthoxeas) and their sizes. Acanthostrongyles, well represented in the spicular complement, are an exclusive trait of the new species widening the morphological range of the genus. Summarizing on spicular complement and spicular morphotraits of 36 species belonging to the genus *Agelas*: i) 32 species show only acanthostyles from Indo-Pacific (n = 14), Atlantic (n = 17), and Mediterranean (n = 1); ii) three Indo-Pacific species show acanthostyles and acanthoxeas; iii) one species *Agelas sansibarica* **sp. n.** from the western Indian Ocean is characterised by the unique trait of three categories of verticillate spicules (acanthostyles, acanthostrongyles and acanthoxeas). A key for the Indo-Pacific species is supplied together with short descriptions, illustrations, and geographic range; literature on chemical bioprospecting of the genus *Agelas* is also provided.

## Introduction

The sponge fauna of the Zanzibar Archipelago is poorly studied and data are reported almost exclusively in very old papers (Lendenfeld 1897, Baer 1906, Jenkin 1908, Sollas 1908, Thomas 1976). In none of these papers species belonging to the genus *Agelas* Duchassaing & Michelotti, 1864 (Porifera: Demospongiae: Agelasida: Agelasidae) are reported. The presence of *Agelas mauritiana* (Carter, 1883) along the Zanzibar coasts was recently recorded (Said et al. 2010) as producer of bioactive compounds.

The widespread genus *Agelas*, including until now 35 valid species, was established by Duchassaing and Michelotti (1864: 76) describing the type species *Agelas dispar* from the Caribbean Sea. *Agelas oroides* is the only Mediterranean species, and is endemic. The western Atlantic (Gulf of Mexico, Caribbean, and Brazil) hosts 17 species. The majority of the latter (13) were recently revised while the remaining four species were considered dubious or suggested as synonyms (Van Soest 2002, Muricy et al. 2011, Parra-Velandia et al. 2014).

The Indo-Pacific species of *Agelas* number 18, including the new species here described. The most widespread species is *Agelas mauritiana* (including its *oxeata* variety) recorded in the Australian western Pacific, and the Indian Ocean from the Mascarenes Archipelago (type locality), Seychelles Archipelago, Madagascar, and Mozambique Channel to the southern Red Sea and east to Sri Lanka.

Several species (14) are reported only once from the type locality i.e. *Agelas axifera* Hentschel, 1911; *Agelas bispiculata* Vacelet, Vasseur & Lévi, 1976; *Agelas braekmani* Thomas, 1998 (1997); *Agelas carpenteri* (Gray, 1867); *Agelas cavernosa* Thiele, 1903; *Agelas citrina* Gotera & Alcolado, 1987; *Agelas dendromorpha* Lévi, 1993; *Agelas inaequalis* Pulitzer-Finali, 1986; *Agelas linnaei* de Voogd, Parra-Velandia & Van Soest, 2008; *Agelas nakamurai* Hoshino, 1985; *Agelas nemoechinata* Hoshino, 1985; *Agelas novaecaledoniae* Lévi & Lévi, 1983; *Agelas robusta*, Pulitzer-Finali 1982; *Agelas semiglaber* Pulitzer-Finali, 1996.

In the framework of sponges, applied research on bioactive compounds at a global level (e.g. Murray et al. 2013) focuses on *Agelas* species as producers of interesting molecules e.g. *Agelas clathrodes*, *Agelas linnaei*, *Agelas mauritiana*, *Agelas nakamurai*, *Agelas oroides*, and *Agelas sceptrum* (Walker et al. 1981, Fathi-Afshar et al. 1989, Keifer et al. 1991, Braekman et al. 1992, Bernan et al. 1993, König and Wright 1993, Chanas et al. 1996, König et al. 1998, Eder et al. 1999, Assmann et al. 2000, 2001, 2004, Fattorusso and Taglialatela-Scafati 2000, Assmann and Köck 2002, Fujita et al. 2003, Bickmeyer et al. 2004, 2005, 2008, Bickmeyer 2005, Costantino et al. 2006, Meketa and Weinreb 2006, Ferretti 2006, Vik et al. 2006, Ding et al. 2007, Ferretti et al. 2007, 2009, Vergne et al. 2008, Hertiani et al. 2010, Said et al. 2010, Regalado et al. 2011, Mordhorst et al. 2015). In this scenario of intensive bioprospecting, research knowledge of systematics and taxonomy in depth is a key tool to identify and define the status of specimens/biomaterial to be processed.

The present paper aims to report the discovery of a new species of *Agelas* from the Zanzibar Archipelago comparing it to all species belonging to the genus. To support global sharing of information on faunistics and taxonomy of Porifera from not widely accessible data sources an updated overview on the morphology and geographic distribution of *Agelas* species from the Indo-Pacific area is also provided together with a brief description and exhaustive iconography, as well as a dichotomous key to Indo-Pacific species.

## Materials and methods

Representative fragments of six sponge specimens from the Unguja Island were studied. Growth form, surface traits, skeletal architecture, shape and size of the spicules are considered diagnostic morphotraits. Spicule dimensions are given as maximum, minimum, and medium lengths and widths of *ca.* 400 spicules.

The skeletal architecture was investigated by hand-cut sections of the ectosome and choanosome. To study the three-dimensional arrangements of fibres and spicules in the skeleton, fragments *ca.* 10 × 10 × 2 mm were cut, cleaned with 5% sodium hypochlorite (NaClO) for 24 h in a warm temperature (35–40 °C), then washed and stirred five times in abundant double distilled water, washed and stirred twice in ethanol 95%, and finally allowed to air dry and gold-sputtered or mounted in Eukitt. The skeletal samples were than studied by light microscope (LM) and scanning electron microscope (SEM). Spicule preparations were made by dissolving a small fragment of the specimen in 65% boiling nitric acid (HNO_3_)and cleaned spicules were rinsed four times with water, once with 95% ethanol. The spicules were air-dried on slides, mounted in Eukitt, and observed by a Leitz Dialux 20 EB (LM), as well as on aluminium stubs and coated with gold for the observation with a Vega3 TESCAN type LMU (SEM).

Specimens were deposited at the Museo civico di Storia Naturale “Giacomo Doria” of Genoa, Italy (acronym MSNG). For the taxonomy of genus and species level the Systema Porifera (Hooper and Van Soest 2002) and the World Porifera Database (Van Soest et al. 2015) are considered as reference.

## Systematic account

**Phylum Porifera Grant, 1835**

**Class Demospongiae Sollas, 1885**

**Order Agelasida Hartman, 1980**

**Family Agelasidae Verrill, 1907**

**Genus *Agelas* Duchassaing & Michelotti, 1864**

***Chalinopsis* Schmidt, 1870** (junior synonym)

***Ectyon* Gray, 1867** (junior synonym)

***Oroidea* Gray, 1867** (junior synonym)

***Pachychalinopsis* Schmidt, 1880** (nomen nudum)

***Siphonochalinopsis* Schmidt, 1880** (nomen nudum)

**Diagnosis** (emended from Van Soest 2002, p. 820). Massive-lobate, encrusting, tubular, branching or flabellate sponges, often of considerable size, with smooth to finely conulose surfaces provided with small rounded and/or key-hole shaped apertures. Colour usually orange or brownish-orange. Consistency toughly compressible, firm. No ectosomal specialization. Choanosomal skeleton isotropic or anisotropic, occasionally irregular, network of primary ascending spongin fibres and secondaries. Main fibres mostly cored by megascleres. Main and interconnecting fibres echinated by megascleres in most cases. Spicules as verticillate styles, or styles and oxeas, or styles, oxeas and strongyles. Biogeographic pattern of 36 nominal species mostly matches tropical waters, a single species occurs in the Mediterranean. The genus has not been recorded from the eastern Pacific, West Africa, and the northern Atlantic European coasts.

### Agelas sansibarica

Taxon classificationAnimaliaAgelasidaAgelasidae

Perino & Pronzato sp. n.

http://zoobank.org/7F8E3929-DD8D-4991-A8E0-C391C89AC1D0

**Figure 1.**
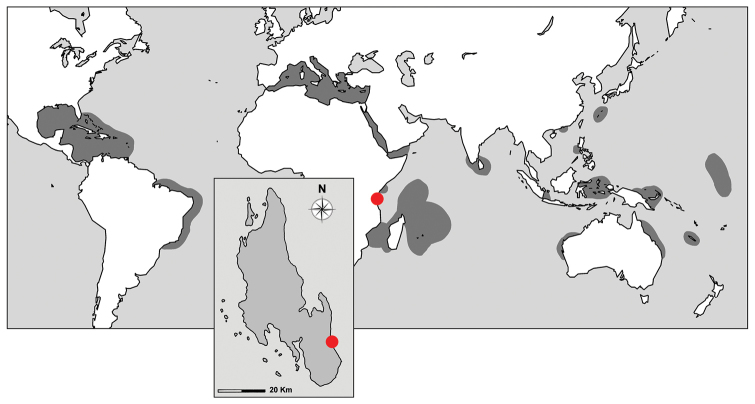
Genus *Agelas*. Biogeographic pattern (grey areas). The red dot indicates the type locality of the new species *Agelas sansibarica* sp. n. at Jambiani (06°18'44.8"S, 39°33'32"E), eastern coast of Unguja Island, Zanzibar Archipelago, United Republic of Tanzania.

**Figure 2.**
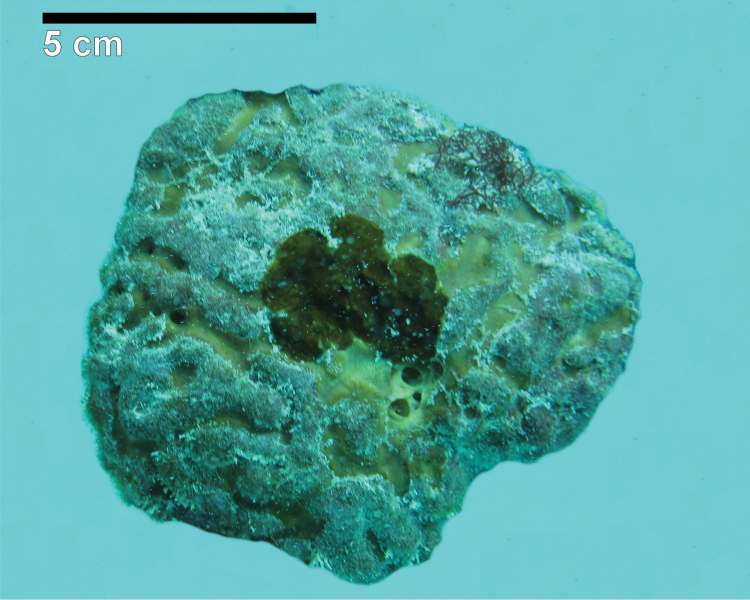
*Agelas sansibarica* sp. n. Type specimen (alcohol preserved, colour different from *in vivo*) from the Zanzibar Archipelago.

**Figure 3.**
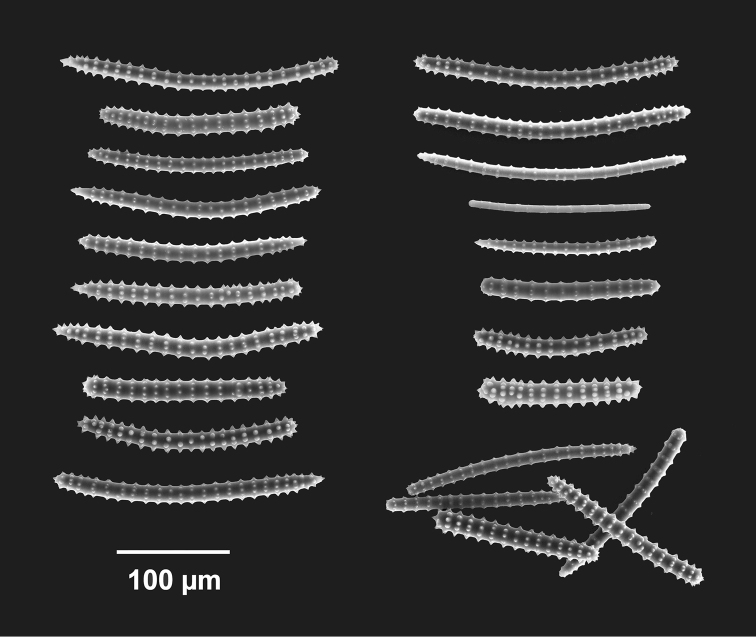
*Agelas sansibarica* sp. n. Spicular complement of verticillate acanthostyles, acanthoxeas and acanthostrongyles (SEM).

**Figure 4.**
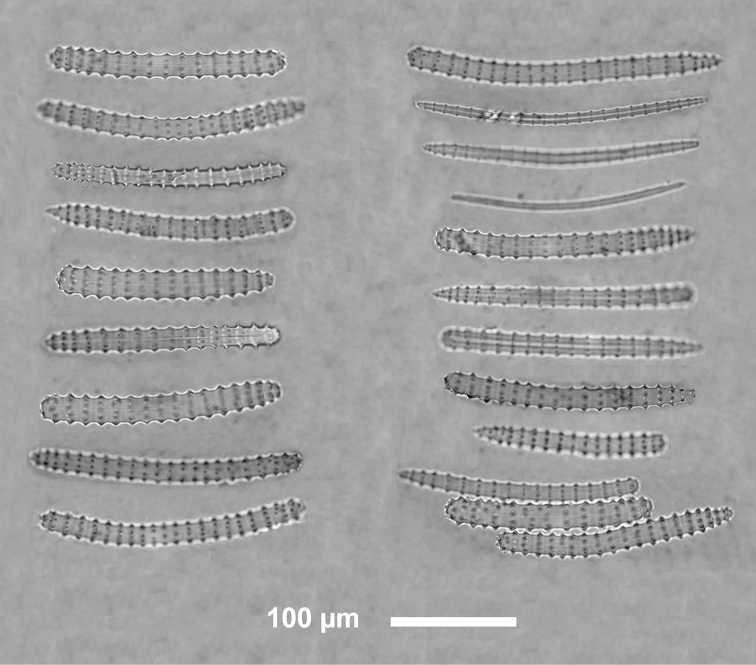
*Agelas sansibarica* sp. n. Spicular complement of verticillate acanthostyles, acanthoxeas and acanthostrongyles (LM).

**Figure 5.**
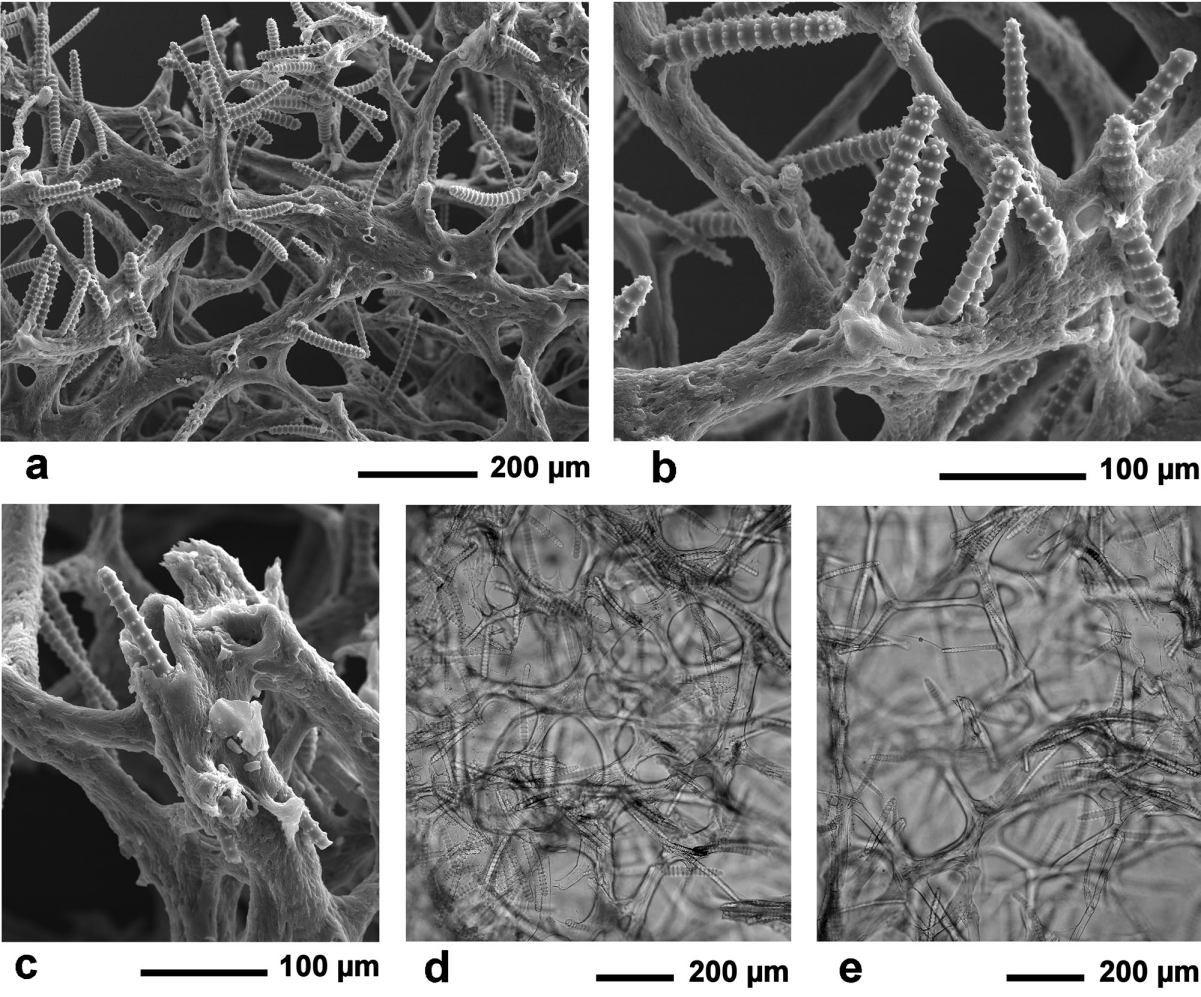
*Agelas sansibarica* sp. n. **a** skeletal network of spongin fibres echinated by spicules (SEM) **b** detail of fibres surface echinated by verticillate spicules (SEM) **c** section of a primary fibre cored by a verticillate acanthostrongyle **d–e** skeletal network (LM).

Figs 1 , 2 , 3 , 4 , 5

#### Material examined.

Holotype: MSNG 57991 (A30), 70% ethanol, Jambiani (06°18'44.8"S, 39°33'32"E), eastern coast of Unguja Island, Zanzibar Archipelago, United Republic of Tanzania, SCUBA diving, 4.vii.2010, leg. Mr. Okala. Paratypes: MSNG 57992, MSNG 57993, MSNG 57994, MSNG 57995, MSNG 57996 (A12, A26, A27, A28, A29, respectively) ibid.

#### Diagnosis.

*Agelas* with unique spicular complement composed of three spicular categories, oxeas, styles and strongyles with spines arranged in a variable number of verticilles.

#### Etymology.

The speciphic epithet refers to the Zanzibar Archipelago.

#### Habitat.

Coral reef, quite common at 7–12 m of depth. Water temperature 28–31 °C. Salinity 20–36‰ (Fryday 2011). As reported by the Swiss Marine NGO manager of the local sponge farming facility (Jambiani Lagoon) the new species is massively farmed (Christian Vaterlaus, pers. comm., 2010).

#### Geographic distribution.

Western Indian Ocean, but only recorded from the type locality to date.

#### Description.

Growth form massive, thick, rounded, 6–10 cm in diameter. Colour in life purple to orange and light brown. Consistency firm and elastic. Surface rough to the touch, finely hispid, finely conulose for tips of ascending fibres supporting the dermal membrane, with regularly scattered circular and convoluted depressions (0.5 cm in diameter) very similar to those of *Agelas cerebrum*. Oscules few, small, irregularly scattered. Choanosomal skeleton as an irregularly reticulate network of spongin fibres. Primary fibres 50–110 (71.67 ±17.63) μm in diameter, strongly echinate by single, scattered spicules to groups of diverging spicules; ascending primary fibres cored by spicules also present. Secondary fibres 20–50 (35 ± 9) μm in diameter notably echinate and cored by spicules. Tertiary network not observed.

Megascleres as three categories of monaxons mostly with acute spines. Acanthostyles 90–250 (180.72 ± 28.66) × 7.5–20 (13.46 ± 2.59) μm ornate by verticillate spines arranged as 11–27 (17.8 ± 2.86) whorls. Acanthoxeas 130–295 (195 ± 43.09) × 7.5–15 (12.17 ± 1.89) μm ornate by verticillate spines arranged as 14–26 (19.24 ± 3.47) whorls. Acanthostrongyles 80–245 (148.18 ± 36.82) × 4–17 (11.09 ± 4.24) μm ornate by verticillate spines arranged as 9–26 (15.76 ± 3.85) whorls. Annulate spicules apparently young.

#### Remarks.

The new species is characterized by the co-presence of three categories of spicules never recorded in other *Agelas* species. Acanthostrongyles are abundant, *ca.* 20–30 % of the total number of spicules.

## Discussion

### Geographic range of Indo-Pacific *Agelas* species

Madagascar, Mozambique Channel, Seychelles and Mascarene archipelagos (Western Indian Ocean province) harbour four species, whereas Japan (Ryukyu Archipelago) and New Caledonia enumerates two species each. Only one species each is recorded from Philippines, Papua New Guinea, and Funafuti. Only one species each is harboured in the Red Sea/Gulf of Aden, Sri Lanka, Moluccas, Sunda Shelf/Java Sea (Indonesia), Hong Kong, Funafuti, and Australia (Fig. 1).

### Diagnostic morphotraits comparative analysis of *Agelas* Indo-Pacific species

To discriminate between all 36 *Agelas* species by diverging diagnostic morphotraits is notably difficult, as highlighted in the previous section. Morphotraits of the genus are extremely conservative and different species appear very similar. Focusing on the Indo-Pacific species our attempt was not as completely successful as is also the case for the Atlantic species by Parra-Velandia et al. (2014).

Atlanto-Mediterranean *Agelas* species (18) seems to possess only acanthostrongyles, including the uncertain *Agelas fascicularis*, *Agelas flabelliformis*, *Agelas inaequalis*, and *Agelas rudis* not redescribed by Parra-Velandia et al. (2014).

Among the 17 previously known Indo-Pacific *Agelas* species, the spicular complement of 14 species is composed of acanthostyles in a single or two-dimensional classes (see Appendix 1, Figs 6–21 for details).

**Figure 6.**
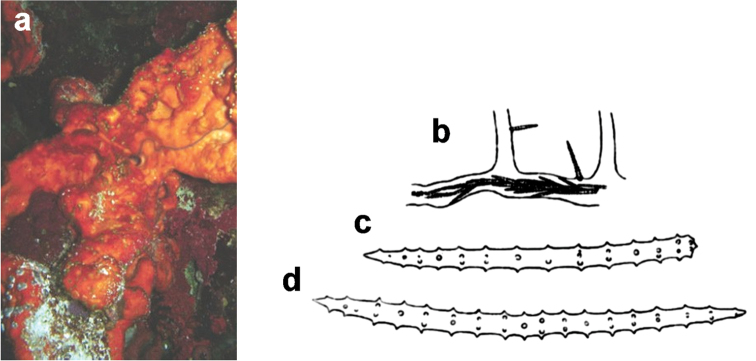
*Agelas axifera*. **a** living specimen **b** skeleton fragment with two spicular types, axially embedded in a fibre and arming the surface **c** acanthostyles **d** acanthoxea (**a** modified from an original underwater shot by J. Hooper **b–d** modified from Hentschel 1911).

**Figure 7.**
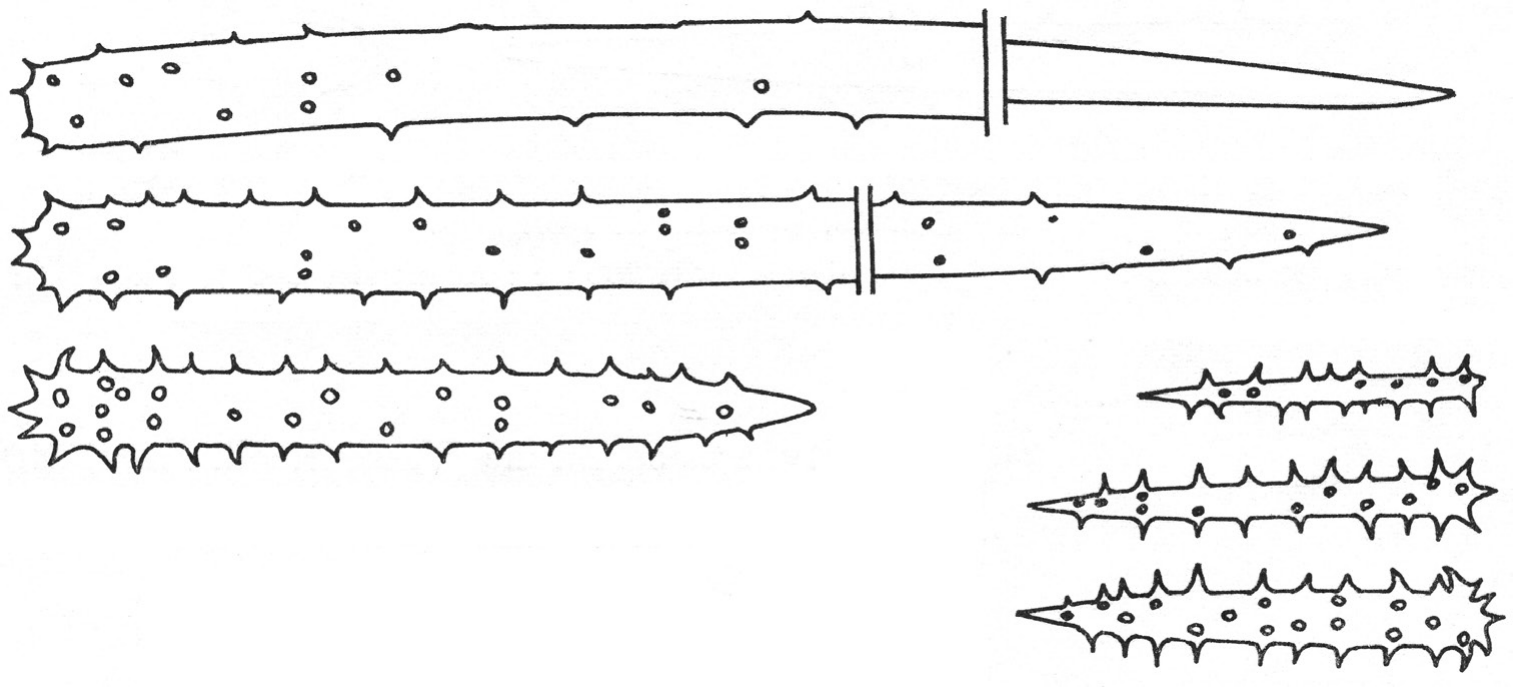
*Agelas bispiculata*. Spicular complement of acanthostyles of two size categories (modified from Vacelet et al. 1976).

**Figure 8.**
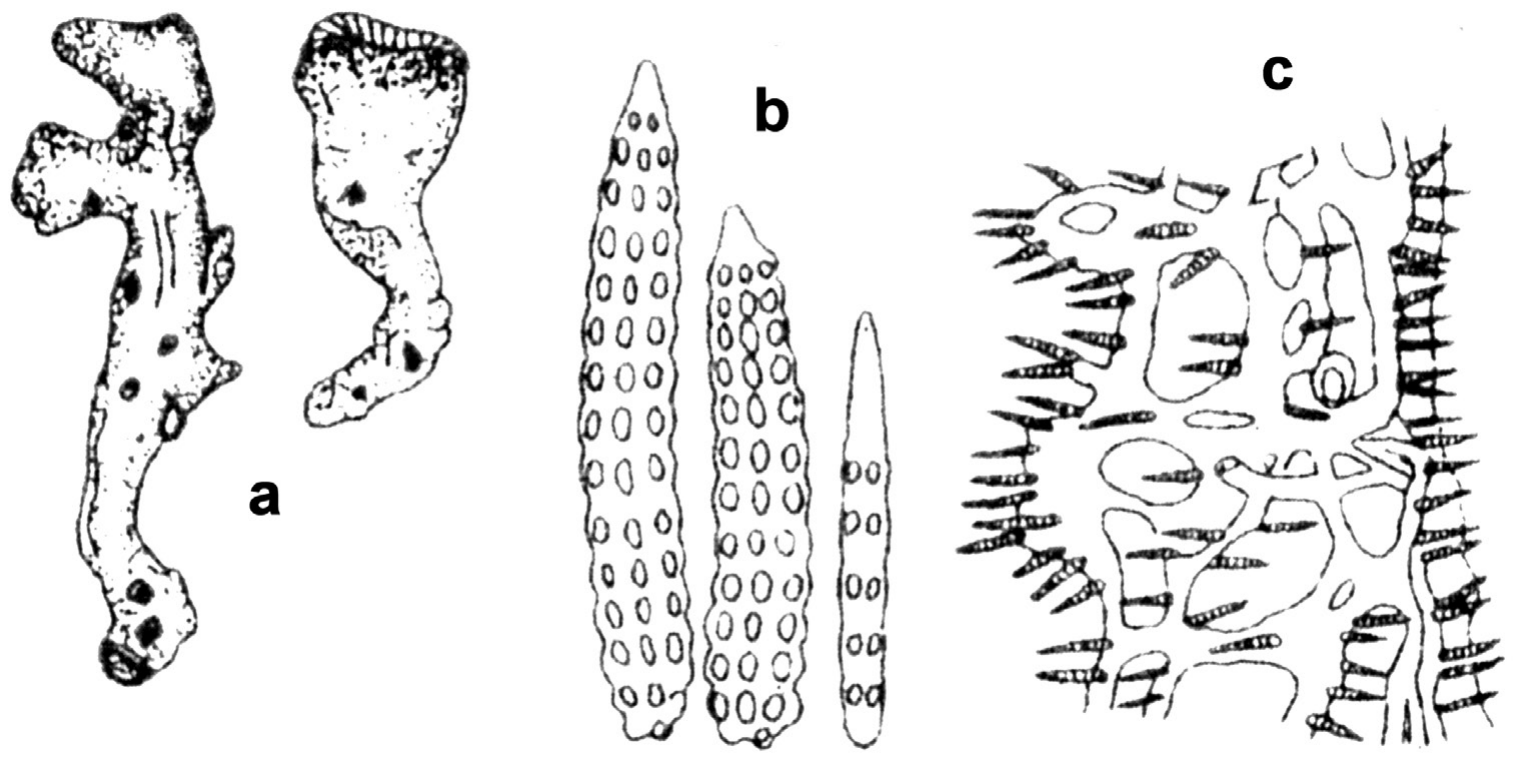
*Agelas braekmani*. **a** schematic drawings of two specimens **b** spicular complement of verticillate acanthostyles **c** skeleton architecture with echinate fibres, sponge surface on the right (modified from Thomas 1998).

**Figure 9.**
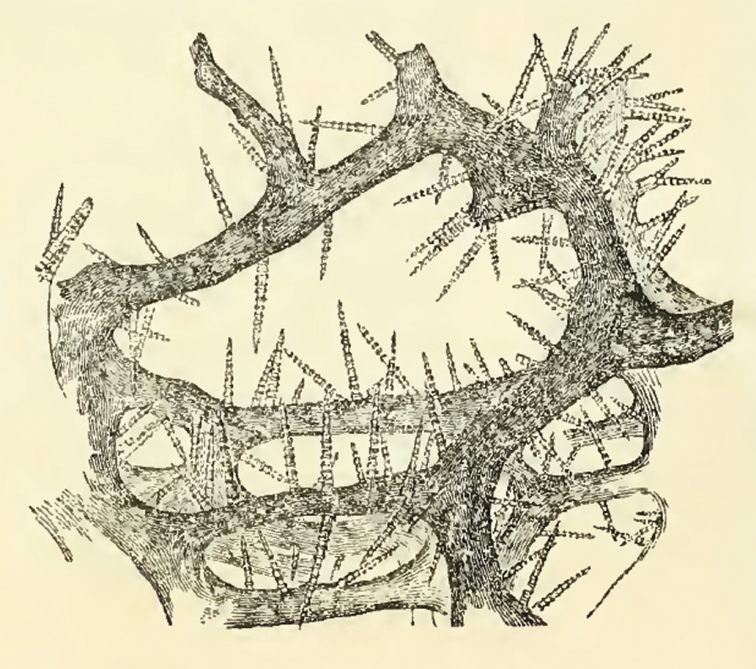
*Agelas carpenteri*. Drawing of the reticulate network with spongin fibres echinated by verticillate spicules perpendicularly arranged (modified from Carpenter 1856).

**Figure 10.**
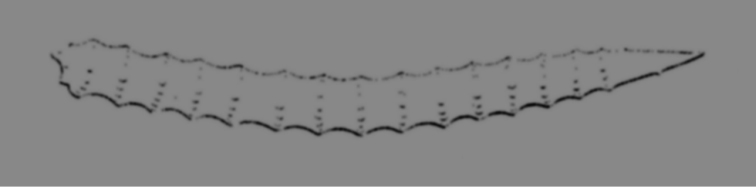
*Agelas cavernosa*. The original illustration of verticillate acanthostyles ornate by spiny whorls by Thiele (1903).

**Figure 11.**
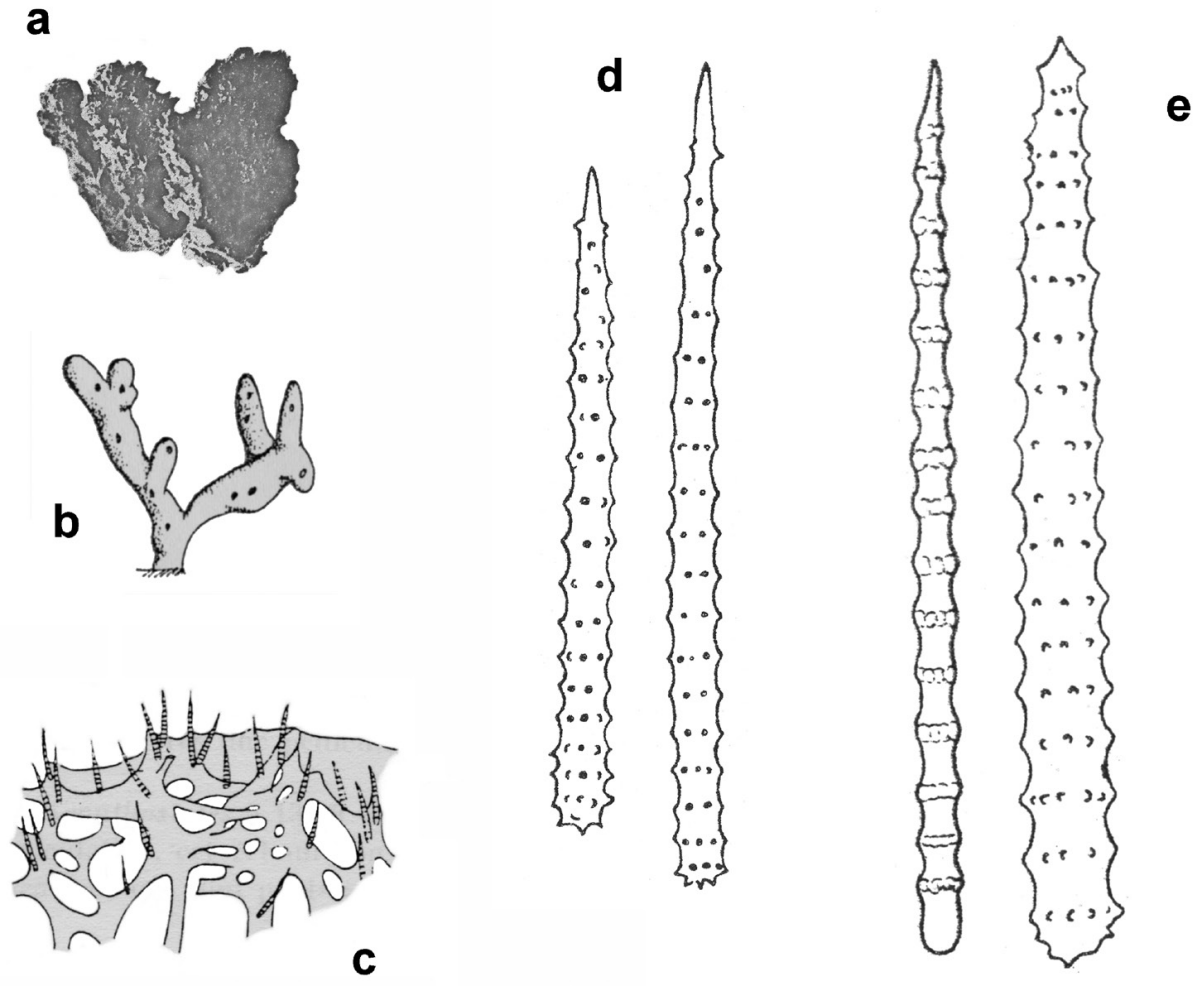
*Agelas ceylonica*. **a** very low quality image of the specimen studied by Dendy 1921 **b** schematic drawing of a branched specimen by Van Soest 1989 **c** skeleton architecture with echinate fibres **d–e** spicular complement of verticillate acanthostyles (**c** modified from Thomas 1981 **d** modified from Dendy 1921 **e** modified from Lévi 1961).

**Figure 12.**
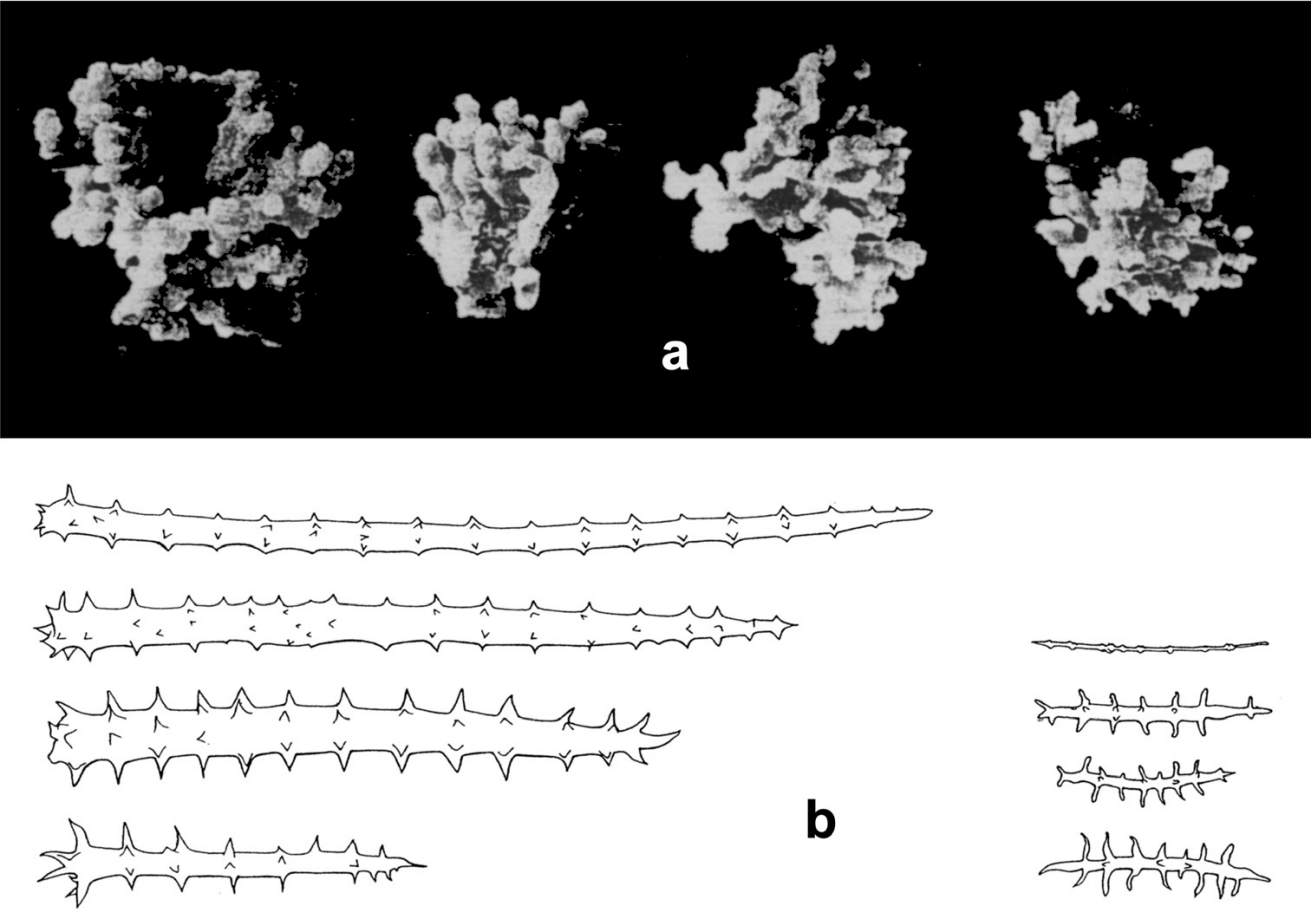
*Agelas dendromorpha*. **a** the sponge specimens of the type series **b** spicular complement of two spicular types, acanthostyles and acanthoxeas; small oxea-like spicules (bottom, right) are not reported in the original description (modified from Lévi 1993).

**Figure 13.**
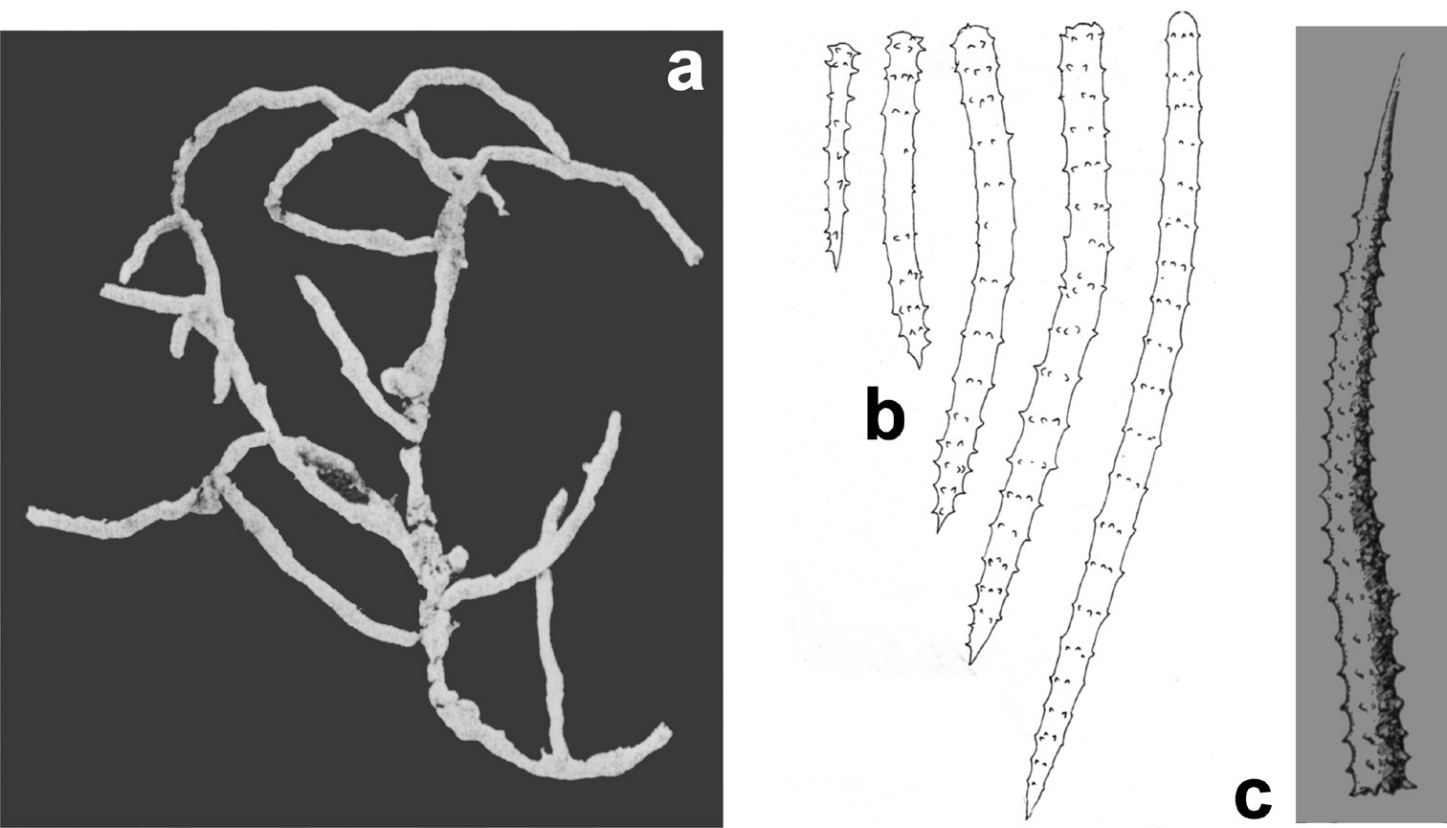
*Agelas gracilis*. **a** ramose slim dry specimen **b**–**c** spicular complement of verticillate acanthostyles of different dimensional categories (**a–b** modified from Lévi and Lévi 1983 **c** modified from Whitelegge 1897).

**Figure 14.**
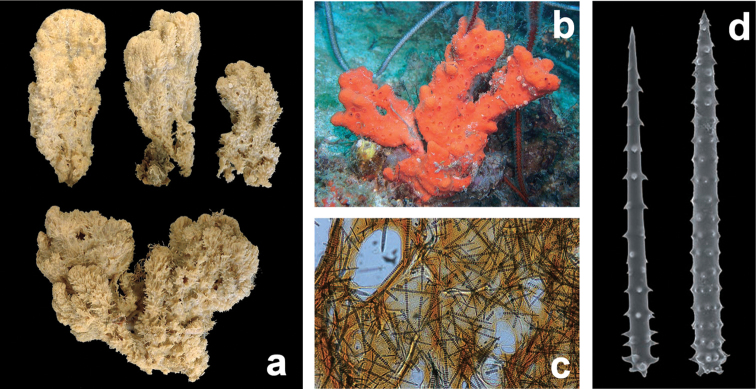
*Agelas linnaei*. **a** type series specimens (liquid preserved) **b** a living shallow water specimen **c** spongin skeleton with spicules **d** verticillate acanthostyles (modified from de Voogd et al. 2008).

**Figure 15.**
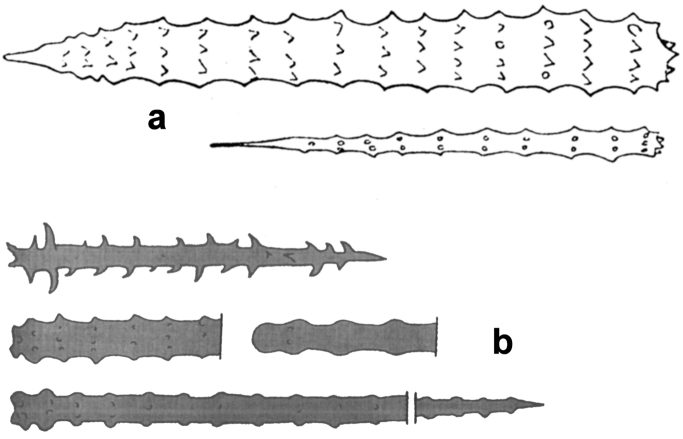
*Agelas marmarica*. **a**–**b** spicular complement (**a** modified from Vacelet and Vasseur 1965 **b** modified from Lévi 1958).

**Figure 16.**
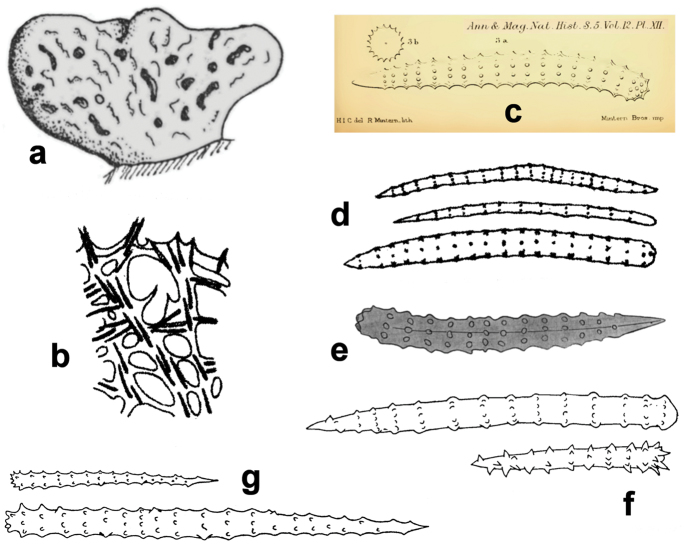
*Agelas mauritiana*. **a** drawing of a massive specimen **b** skeleton fragment **c**–**g** spicular complement **b–d** Agelas mauritiana var. oxeata **a** modified from Van Soest 1989; **b**–**d** modified from Thomas 1979 **c** modified from Carter 1883 **e** modified from Vacelet and Vasseur 1965 **f** modified from Lévi 1964 **g** modified from Lévi 1961).

**Figure 17.**
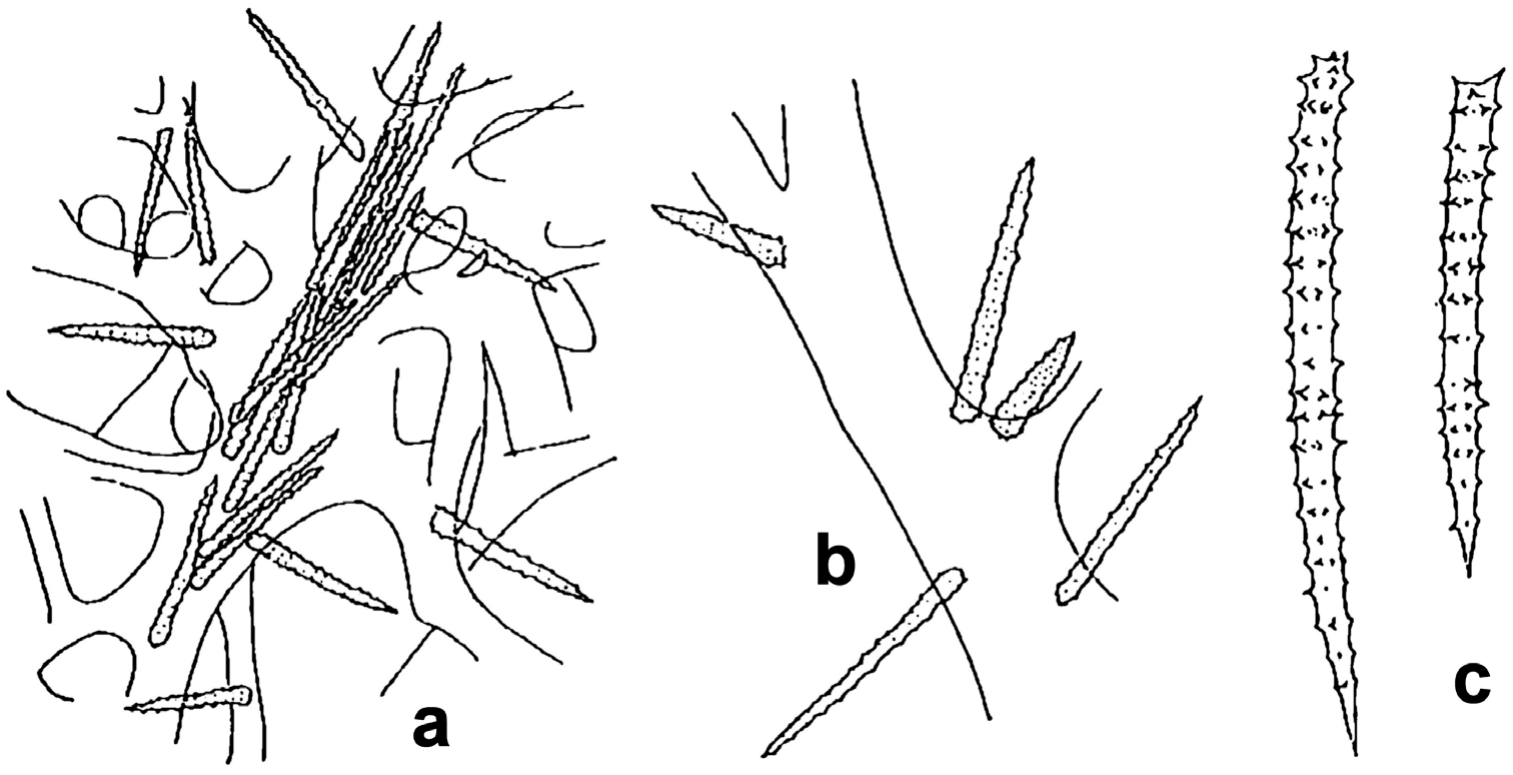
*Agelas nakamurai*. **a** skeleton architecture **b** close up of spicules insertion in the spongin fibres **c** acanthostyles (modified from Hoshino 1985).

**Figure 18.**
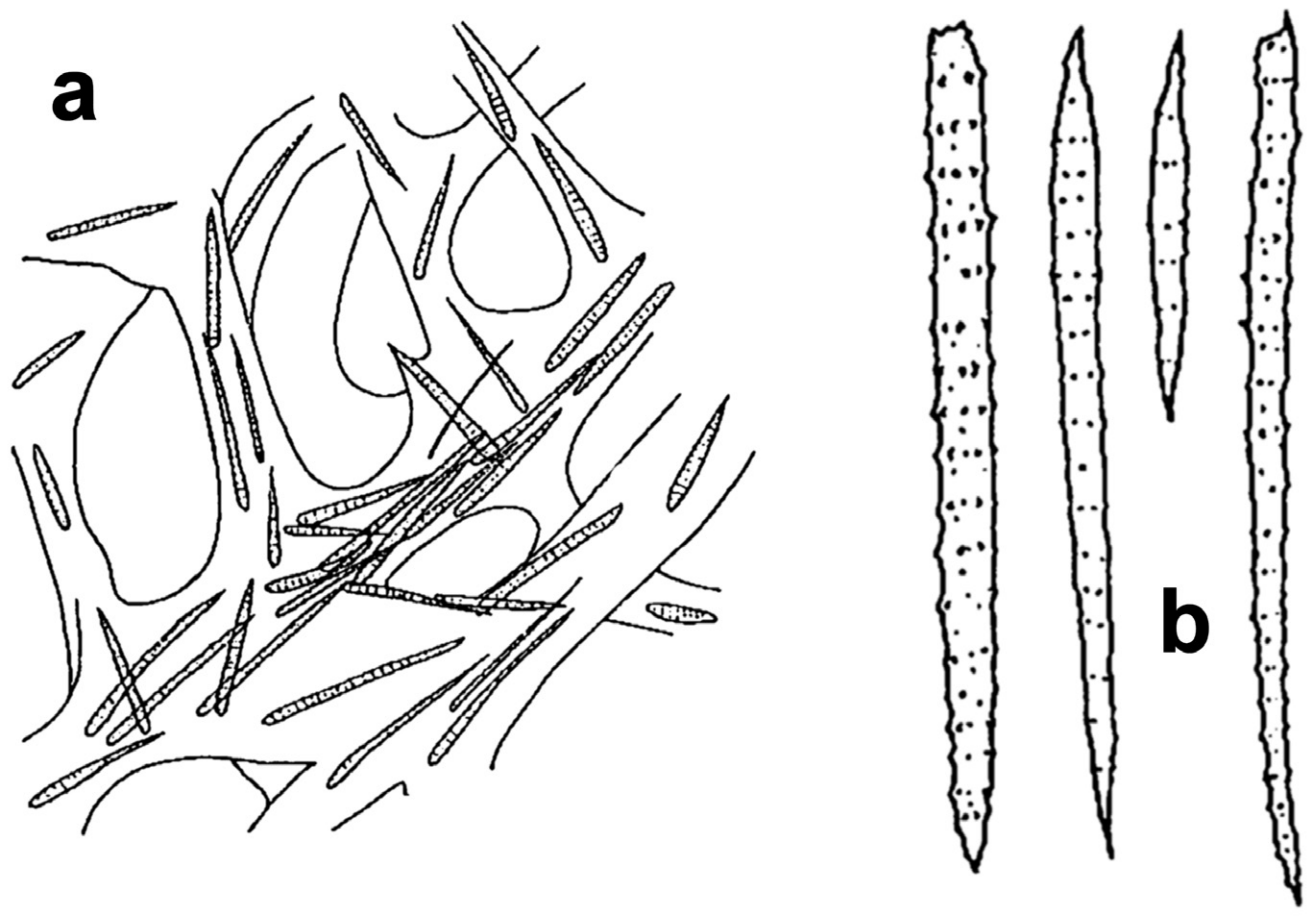
*Agelas nemoechinata*. **a** skeleton architecture **b** spicular complement (modified from Hoshino 1985).

**Figure 19.**
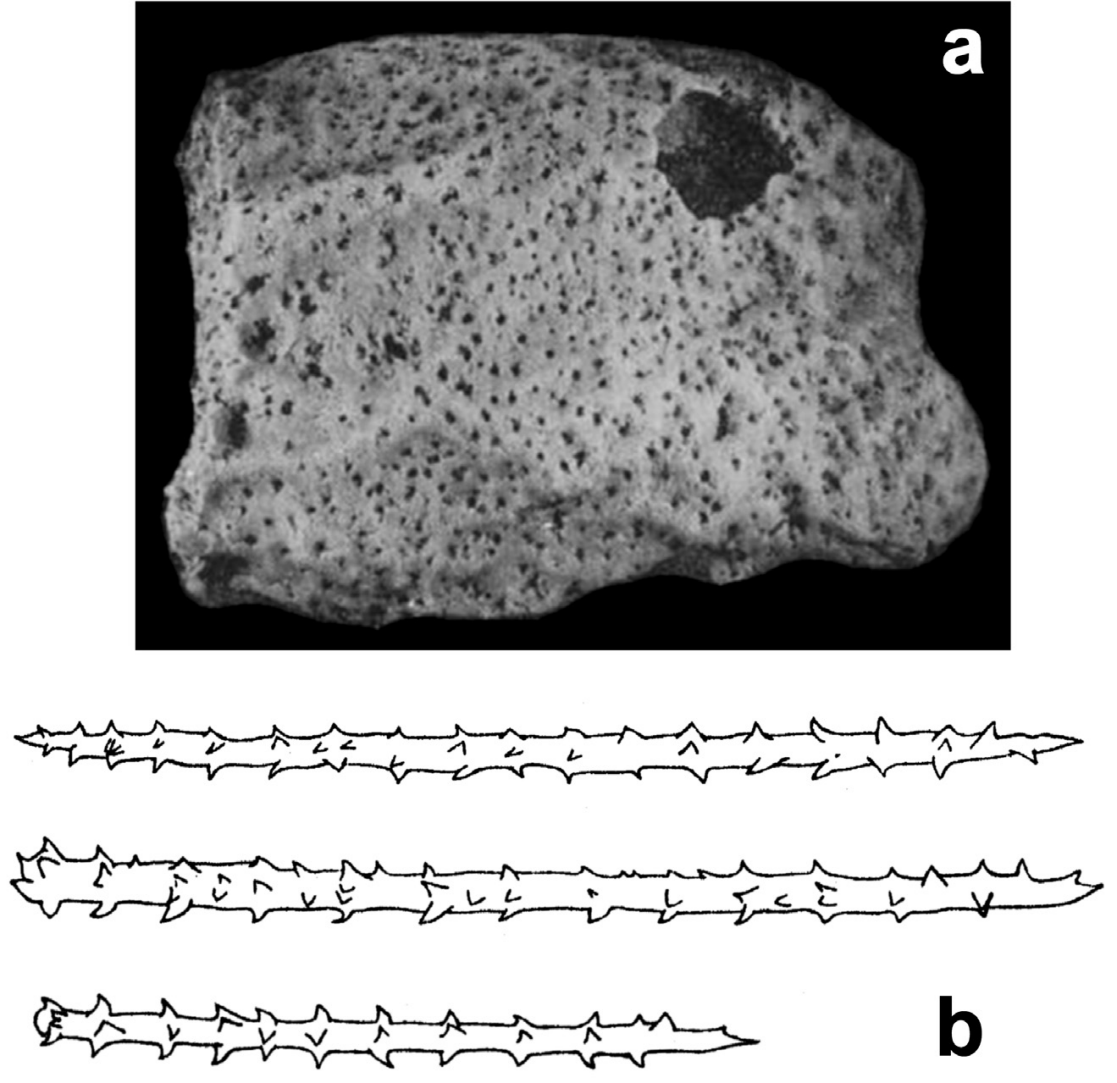
*Agelas novaecaledoniae*. **a** type specimen **b** spicular complement with two spicular types (modified from Lévi and Lévi 1983).

**Figure 20.**
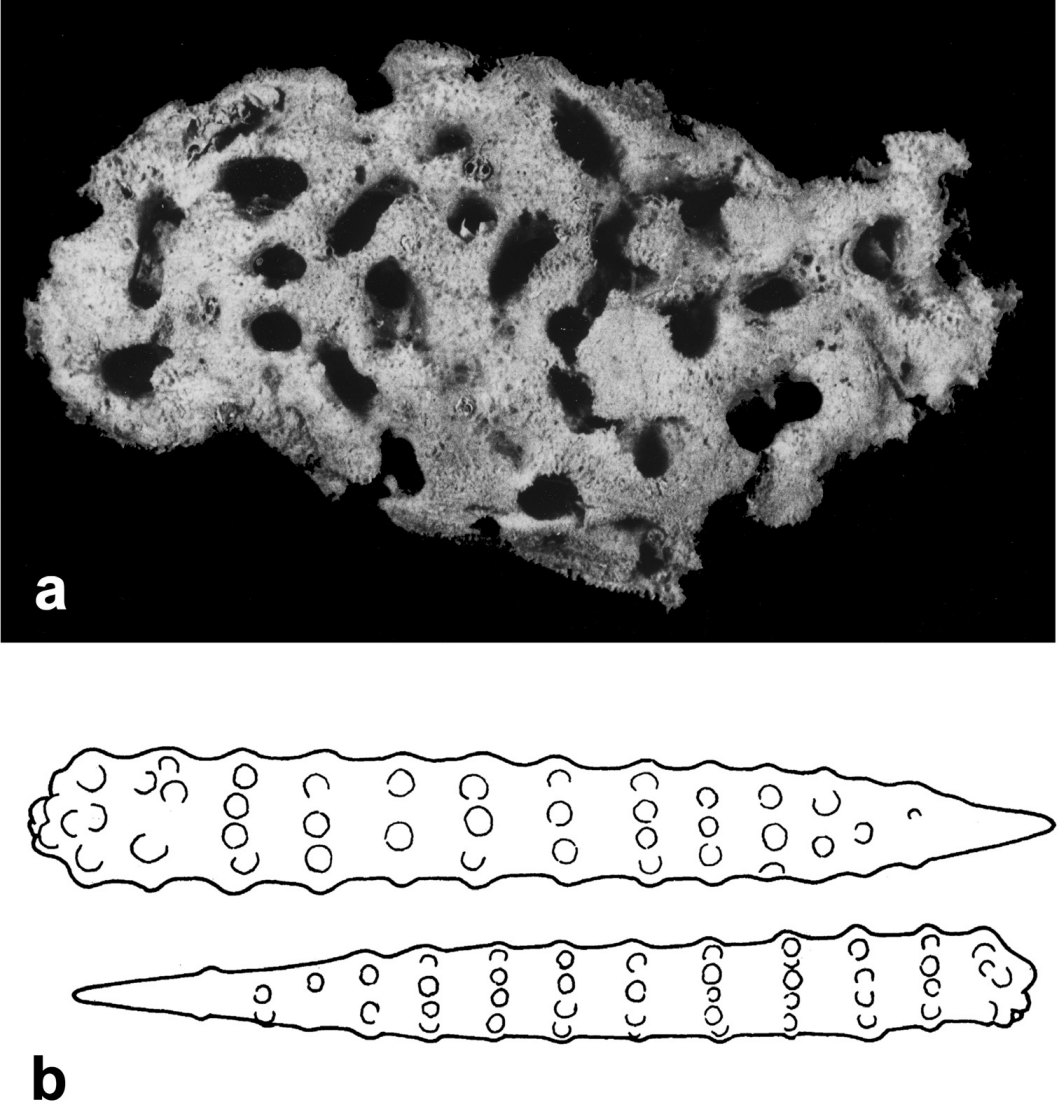
*Agelas robusta*. **a** type specimen (dry) **b** spicular complement of acanthostyles very stout, verticillate by blunt spines (modified from Pulitzer-Finali 1982).

**Figure 21.**
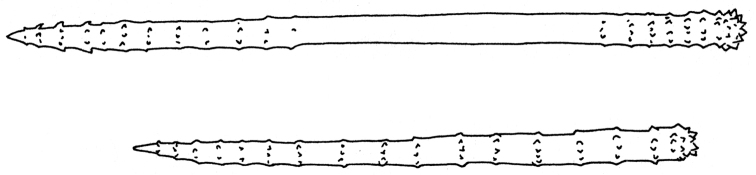
*Agelas semiglabra*. Spicular complement with two dimensional categories of acanthostyles; long acanthostyles spiny only at the tips (modified from Pulitzer-Finali 1996).

The most common and studied Indo-Pacific species, i.e. *Agelas mauritiana*, are characterized by a single spicular type acanthostyles, which are extremely variable in morphology, abundance of spines, and dimensional range (sometime more than three times in length) (see Table 1). The Atlantic *Agelas dispar* and the Mediterranean *Agelas oroides* show a similar size variability of acanthostyles. Only the Indo-Pacific *Agelas axifera*, Agelas mauritiana var. oxeata, and *Agelas novaecaledoniae* show two different categories of spicules, i.e. acanthostyles and acanthoxeas.

**Table 1.** *Agelas mauritiana*. Morphometries and morphotraits by different authors.

References	Acanthostyles μm	Whorls nº	Colour	Habitus size (cm)	Consistency
Carter 1883	132	15–18	-	-	-
Thiele 1903	200 × 14–15	16	-	-	-
Dendy 1905	176 × 16	–	dark brown	tubular 3.1 × 1.6 length × diameter	firm resilient
Laubenfels 1954	170–180 × 10–14	12–18	-	-	-
Lévi 1961	150–160 × 8–12	16–20	brown	massive 6–10 × 4	firm resilient soft
Lévi 1964	275 × 12–13	15–17	-	-	-
Lévi 1967	140–230 × 8–10	10–15	-	-	-
Vacelet and Vasseur 1965	135–250	13–20	-	-	-
Vacelet and Vasseur 1971	80–180 × 7.5–12.5	18–23	brown	encrusting 1.6–1.5 thickness	firm resilient coriaceus
Thomas 1979	112–212 × 6–8	6–8	pale yellow	encrusting cavernous	firm resilient compressible

The new species *Agelas sansibarica* is characterized by the unique morphotrait of three categories of megascleres, i.e. acanthostyles, acanthoxeas, and acanthostrongyles with spines arranged in verticilles regularly scattered along the entire spicule. No other *Agelas* species exhibit this spicular combination. Acanthostrongyles, well identifiable and abundant, represent an exclusive diagnostic trait of the new species. The functional role of acanthostrongyles is doubled since echinanting spicules arm both the fibres surface and the core of the axial part of fibres.

Summarizing spicular complements and spicular morphotraits of 36 *Agelas* species: i) 32 species show only acanthostyles from Atlantic (17), Mediterranean (1), and the majority (14) of the Indo-Pacific areas; ii) three Indo-Pacific species show acanthostyles and acanthoxeas; iii) only one species *Agelas sansibarica* sp. n. from the western Indian Ocean show a spicular component composed of acanthostyles, acanthoxeas, and acanthostrongyles.

### Key to the Indo-Pacific *Agelas* species

The present key is an attempt to discriminate between the Indo-Pacific species, but the scenario appears very intricate mainly because morphotraits from many descriptions and illustrations are overlapping (see Table 1). A similar attempt, on the Atlantic species, was performed by Parra-Velandia et al. (2014) emphasizing that: “Caribbean *Agelas* taxonomy rests heavily on the external morphology”; as a consequence their key is essentially based on growth form and colour. Since this is the situation, our key is not simply dichotomous and allows the disctintion of only 13 of the 17 Indo-Pacific species (see Appendix 1). The remaining four species have acanthostyles with length ranges which are widely overlapping (from less than 150 to more than 250 μm). Three of these (*Agelas carpenteri*, *Agelas marmarica*, and *Agelas robusta*) are known only from the original descriptions; on the contrary, *Agelas mauritiana* is reported by several authors but with discordant descriptions (Table 1).

**Table:** 

1	Spicular complement composed by 1 or 2 spicular types (acanthoxeas, acanthostyles)	**2**
–	Spicular complement composed by 3 spicular types (acanthoxeas, acanthostyles, acanthostrongyles)	***Agelas sansibarica* sp. n.**
2	Spicular complement composed by 2 spicular types (acanthoxeas and acanthostyles)	**3**
–	Spicular complement composed by 1 spicular type (acanthostyles)	**4**
3	Sponge body cup-shaped	***Agelas axifera***
–	Sponge body blade-shaped	***Agelas novaecaledoniae***
–	Sponge body lobed	***Agelas mauritiana oxeata***
–	Sponge body digitate	***Agelas dendromorpha***
–	Sponge body as slim cylindrical erected axis (branched or unbranched)	***Agelas gracilis***
4	Acanthostyles of 2-dimensional categories	**5**
–	Acanthostyles of 1-dimensional category	**7**
5	Long acanthostyles spiny only at the tips	***Agelas semiglabra***
–	Long and short acanthostyles almost entirely spiny	***Agelas bispiculata***
7	Primary and secondary fibres uncored	**8**
–	Primary and/or secondary fibres cored	**9**
8	Acanthostyles longer than 300 μm	***Agelas ceylonica***
–	Acanthostyles length no more than 200 μm	***Agelas cavernosa***
9	Primary and secondary fibres cored	***Agelas nemoechinata***
–	Primary fibres cored and secondary uncored	**10**
10	Acanthostyles (185–265 × 8–15 μm) with 15–23 whorls	***Agelas nakamurai***
–	Acanthostyles (130–220 × 4–21 μm) with 8–18 whorls	***Agelas braekmani***
–	Acanthostyles (80–370 × 5–24 μm) with 11–33 whorls	***Agelas linnaei***

## Supplementary Material

XML Treatment for Agelas sansibarica

## Appendix I

Indo-Pacific species belonging to the genus *Agelas* reported from the literature.

### *Agelas axifera* Hentschel, 1911

Fig. 6

**Description** (modified from Hentschel 1911). Growth form club-shaped (12 × 6 cm), walls 0.5 cm in thickness. Surface irregular. Colour orange. Ectosomal skeleton not reported. Choanosomal skeleton irregularly reticulate network (meshes 320 μm in diameter) of fibres echinated by few spicules. Primary fibres (80 μm in diameter) cored by spicules. Secondary fibres (40 μm in diameter) not cored. Megascleres verticillate of two categories. Acanthostyles and acanthoxeas of similar size (112–152 × 5–7 μm) ornate by 7–15 regular whorls with 5–6 acuminate thorns each one. **Habitat.** Not reported. **Geographic distribution.** Pacific Ocean, Australia. **Remarks.** Type specimen stranded on beach at Champion Bay, Geraldton, Western Australia.

### *Agelas bispiculata* Vacelet, Vasseur & Lévi, 1976

Fig. 7

**Description** (modified from Vacelet et al. 1976). Growth form massive; hemispherical fragments (5–6 cm in diameter). Surface hispid. Oscules and pores not evident. Consistency elastic. Colour yellowish in spirit. Ectosomal skeleton not reported. Choanosomal skeleton as reticulate network of thick, armed spongin fibres. Primary fibres (70 μm in diameter) axially cored by large acanthostyles type I and echinated by small acanthostyles type II. Secondary fibres (40–50 μm in diameter) less abundant, without spicules. Megascleres of one category, acanthostyles of two dimensional classes. Acanthostyles type I (320–400 × 14–17 μm) ornate by 20 not accentuate whorls. Acanthostyles type II (55–120 × 6–10 μm) ornate by 11–15 accentuate whorls. **Habitat.** Coral reefs, shade zone (caves, cavities). **Geographic distribution.** Western Indian Ocean. Recorded only from Mozambique Channel (eastern Madagascar). **Remarks.** de Voogd et al. (2008) focused on larger acanthostyles localized in the axial skeleton, and the smaller ones echinanting the fibres. Known only from the type locality, Grand Recife, Entsetreky (Tulear), Madagascar.

### *Agelas braekmani* Thomas, 1998

Fig. 8

**Description** (modified from Thomas 1998). Growth form tubular and irregularly branched 10 mm in diameter, branches tips blind or with apical oscule 2–4 mm in diameter, or sometime bearing a funnel-shaped opening with an orifice 30 mm in diameter. Consistency stiff, cork-like. Foreign particles as shell pieces embedded on the wall at place. Surface hispid. Ectosomal skeleton as erected spicules supporting the dermal membrane. Choanosomal skeleton as reticulate network of spiculous spongin fibres, mesh size 180–560 μm. Primary fibres echinate and thick (up to 132 μm in diameter) feeble cored by acanthostyles. Secondaries not cored, fairly common echinated by spicules (42–76 μm). Megascleres verticillate of a single category. Acanthostyles 130–220 (159) × 4–21 μm (15 μm excluding spines; up to 25 μm with spines) ornamented by verticillate spines arranged as 8–18 whorls. Younger spicules partly annulated, each spine tubercled at the base. **Habitat.** Shallow water. **Geographic distribution.** Pacific Ocean. Recorded from Hansa Bay (type locality), Madang Province, Bismarck Sea. **Remarks.** This species was not considered in the revision by de Voogd et al. (2008).

### *Agelas carpenteri* (Gray, 1867)

Fig. 9

*Halichondria* (?) Carpenter, 1856 in Gray (1867)

*Ectyon carpenteri* Gray, 1867

**Description** (modified from Gray 1867). Growth form massive. Ectosomal skeleton not reported. Choanosomal skeleton as a reticulate network of cylindrical spongin fibres echinated by single scattered spicules or groups of diverging spicules. Spicules fusiform, verticillate with *ca.* 10 whorls. **Habitat.** Not reported. **Geographic distribution.** Western Indian Ocean. Recorded only once from Madagascar. **Remarks.** This species is poorly described and illustrated. The spicule typology (oxeas or styles) are not reported by the author. Gray erected this species on the basis of what is shown by Carpenter (1856) in his book on microscopy.

### *Agelas cavernosa* Thiele, 1903

Fig. 10

**Description** (modified from Thiele 1903). Growth form irregularly massive with basal portion bearing digitiform outgrowths with a single, large oscule at the tips. Surface grainy with small stones or other foreign particulate. Colour blackish. Cavernous structure. Ectosomal skeleton not reported. Choanosomal skeleton as a dense network of brownish fibres. Fibres uncored, echinated by spicules. Megascleres of a single category. Acanthostyles (200 × 15 μm) verticillate, ornamented by 16 whorls of 15 spines each. **Habitat.** Tropical; not reported in detail. **Geographic distribution.** Ternate (type locality), Halmahera, Maluku Islands (Moluccas), eastern Indonesia. **Remarks.** The original description and particularly the illustration (only one spicule) are not exhaustive.

### *Agelas ceylonica* Dendy, 1905

Fig. 11

**Description** (modified from Dendy 1905, 1921, Lévi 1961, Thomas 1981). Growth form notably variable from ramose with slender subcylindrical digitiform branches with blunt tips (2–5 mm in diameter; 27 mm total height) to bushy, very irregular 60 × 41 × 40 mm (Lévi 1961) or encrusting (20 × 15 mm, 1–3 mm in thickness) with oscules and pores not evident (Thomas 1981). Surface minutely hispid by spicules, irregular, conulose due to sharp, blunt, minute conules resulting from the protruding tips of ascending fibres. Consistency compressible, quite soft, elastic, resilient, fibrous and fairly tough in spirit. Colour brown, dark brown in spirit (Dendy 1905, 1921), orange (Lévi 1961), pale gray (Thomas 1981). Ectosomal skeleton as erect spicules (Thomas 1981). Choanosomal skeleton irregular network of pale-coloured spongin fibres (30 μm in diameter) cored by spicules and abundantly echinated by acanthostyles. Ascending primary and secondary fibres not well defined (20–60 μm in diameter), never cored by spicules (Thomas 1981). Megascleres asbelonging to two categories of styles: spiny, very variably verticillate and smooth. Acanthostyles 240 × 20 μm (Dendy 1905, 1921), 80 –275 × 5–15 μm with 16–21 verticilles and 100–300 × 6–15 μm with 13–23 verticilles (Lévi 1961), acanthostyles (240 × 2 μm) straight to slightly curved, with small, conic spines (Thomas 1981). Smooth styles (320 × 24 μm) less frequently present (Dendy 1921). **Habitat.** Not reported. **Geographic distribution.** Indian Ocean. Recorded from south India, Sri Lanka, Indonesia, and Seychelles. **Remarks.** Recently recorded from Indonesia (de Voogd et al. 2008). The latter authors report that acanthostyles in the Manaar Gulf (type locality) specimens have a maximum dimension of 240 × 20 μm.

### *Agelas dendromorpha* Lévi, 1993

Fig. 12

**Description** (modified from Lévi 1993). Growth form bush-like (30–35 × 15–40 mm) branched, with main stem 9–10 mm in height, 3 mm in diameter. Branches with terminal buds (2–3 mm in diameter). Dermal membrane hispidate. Ectosomal skeleton as small acanthostyles in the dermal membrane. Choanosomal skeleton dense with spongin fibres echinated by spicules at the end of the main column or branches. Megascleres of a single category with two dimensional classes. Acanthostyles I (130–260 × 10–12 μm) abundant, slightly curved, verticillate by 12–18 irregular whorls. Acanthostyles II (60–100 × 3–4 μm) from the ectosome less abundant, with 8–9 less organized whorls. **Habitat.** Deep water, 245–275 m of depth. **Geographic distribution.** Western Pacific Ocean. Recorded only from the type locality in New Caledonia. **Remarks.** Also small oxea-like spiny spicules reported in original illustrations but not in descriptions. de Voogd et al. (2008) reported that verticillation is absent in smaller acanthostyles.

### *Agelas gracilis* Whitelegge, 1897

Fig. 13

**Description** (modified from Whitelegge 1897, Lévi and Lévi 1989). Growth form subcylindrical, thin (2–3 mm in diameter; 25–75 mm in length) unbranched, settled on shells fragments. Surface uneven, hispid, with numerous minute conules (0.2–0.5 mm in height, 2–5 mm apart). A few minute pores between the conules. Consistency soft, spongy but also a bit tough. Colour greyish-yellow in spirit. Ectosomal skeleton not reported. Choanosomal skeleton network irregularly reticulate of spongin fibres, with oval or oblong mesh, rarely angular. Primary fibres (70 μm in diameter) with an axial plexus from which secondary (45 μm in diameter) and tertiary fibres (25 μm in diameter) are given off. Megascleres of single category. Acanthostyles (100–220 × 7–13 μm) verticillate by 16 to 24 whorls of small spines (Whitelegge 1897). **Habitat.** Coral reef, 72–125 m of depth. **Geographic distribution.** Western Pacific Ocean. Recorded from Funafuti and Philippines. **Remarks.** Lévi and Lévi (1989) reported branched growth form, fibres (20–30 μm in diameter), acanthostyles of two dimensional classes, type I 90–120 × 7–8 μm with 9–12 verticilles, and type II 190–290 × 8–13 μm with 17–21 verticilles. de Voogd et al. (2008) report that the specimen from the deeper subtidal (85–90 m) in the Philippines (Lévi and Lévi 1989) was ascribed to this species despite the divergence in spicular size.

### *Agelas linnaei* de Voogd, Parra-Velandia & Van Soest, 2008

Fig. 14

**Description** (modified from de Voogd et al. 2008). Growth form roundly lobate to thickly flabellate (8 cm in height, 2.5 cm in diameter). Consistency very soft, spongy. Colour bright orange at the surface to cream-orange internally. Surface with dense conules (1–3 mm in height) supported by tips of ascending fibres covered by a bright easily distinguishable membrane. Small apertures (< 2 mm) scattered on the surface, bigger pores (2–3 mm) connected to internal axial canals sometime present between some lobes. Choanosome dense with narrow canals (primary canals 200 μm–2.00 mm in diameter; secondary canals 100 μm–1.00 mm in diameter). Ectosomal skeleton not reported. Choanosomal skeletal network irregularly and densely reticulate; primary fibres (35–80 μm in diameter) aggregated in packs, more or less undulated, heavily cored (1–7 spicules in cross section) and echinate; secondary fibres (25–40 μm in diameter) not cored and less echinate. Megascleres of a single category. Acanthostyles (78.7–(187)–372.3 × 5.2–(12.1)–24 μm) straight, a few slightly curved, ornate by 11–(19.3)–33 whorls with 5–12 spines each; whorls conspicuous in the spicule centre but sometimes faint and irregular at the spicule tip and head. **Habitat.** Coral reef, overgrowing other reef invertebrates. **Geographic distribution.** Recorded from the Thousands Islands Reef complex, off Jakarta, West Java, Indonesia. Type locality: Peniki Island and Payang Island. **Remarks.** de Voogd et al. (2008) focused on the whorl measurements performed in the middle third of the spicule because spine abundance is dependent upon the width. This is a useful rule to perform uniform measurements of whorls in verticillate spicules.

### *Agelas marmarica* Lévi, 1958

Fig. 15

**Description** (modified from Lévi 1958, Vacelet and Vasseur 1965, Vacelet and Vasseur 1971). Growth form not recorded. Colour orange to/or bright red. Ectosomal skeleton not reported. Choanosomal skeleton as a reticulate network of fibres <30 μm in diameter. Megascleres of a single category. Acanthostyles (230 × 10 μm) verticillate by 19–21 whorls of spines (Lévi 1958). Acanthostyles type I 100–270 × 7–14 μm with 21–25 whorls; acanthostyles type II 160–215 × 7–20 μm with 16–24 whorls (Vacelet and Vasseur 1965). **Habitat.** Coral reef, 20–30 m of depth. **Geographic distribution.** Indian Ocean. Recorded from Mozambique Channel (Madagascar) and Red Sea. Type locality: Saudi Arabian Red Sea. **Remarks.** Spicules morph and their sizes are very variable in descriptions and illustrations of different authors.

### *Agelas mauritiana* (Carter, 1883)

Fig. 16, Table 1

*Ectyon mauritiana* Carter, 1883

**Description** (modified from Carter 1883, Thiele 1903, Dendy 1905, Laubenfels 1954, Lévi 1961, 1964, 1967, Vacelet and Vasseur 1965, 1971, Thomas 1979, 1998). Growth form highly variable: encrusting, tubular, massive, sometimes cavernous (Table 1) also ramose or massively branched 4.5 cm in height bearing five blunt branches 2 cm in diameter with a circular oscule at the tip. Surface with raised oscules 2–3 mm in diameter (Lévi 1961, Vacelet and Vasseur 1971). Consistency firm, resilient coriaceus, but also soft, compressible. Colour notably variable (Table 1). Surface distinctly conulose at growing portions, older ones smooth and glabrous (Thomas 1979). Ectosomal skeleton not reported. Choanosomal skeleton as a dense, irregularly reticulate network of stout fibres very abundantly echinated (Dendy 1905, Lévi 1961, Thomas 1979). Primaries and secondary fibres amber -coloured and not differentiated from each other (Thomas 1979). Megascleres as verticillate acanthostyles of two size classes. Acanthostyles 130–275 × 8–20 μm with 10–20 whorls (Table 1). Smaller acanthostyles also reported (Table 1). **Habitat.** Not reported. **Geographic distribution.** Indian Ocean and West Pacific. Mascarene Archipelago (Mauritius is the type locality) and Seychelles Archipelago (Aldabra), Madagascar, Mozambique, Gulf of Aden, southern Red Sea, Gulf of Aqaba, Sri Lanka, and Australia. **Remarks.** Variable morphometries are reported by different authors (Table 1).

### Agelas mauritiana var. oxeata Lévi, 1961

Fig. 16

**Description** (modified from Lévi 1961). Growth form lobed, each of the two lobes measures 20 mm in length and 8 mm in diameter. Colour brownish-red. Surface velvety. Choanosomal skeleton of primaries fibres cored by oxeas. Megascleres (100–220 × 4–18 μm) of two categories. Acanthostyles verticillate with 14–16 whorls. Acanthoxeas verticillate with 20–24 whorls. **Habitat.** Shallow water, 12 m of depth. **Geographic distribution.** Indian Ocean. Recorded only from Aldabra Island (type locality) in the Seychelles Archipelago. **Remarks.** This variety was not considered in the revision by de Voogd et al. (2008).

### *Agelas nakamurai* Hoshino, 1985

Fig. 17

**Description** (modified from Hoshino 1985). Growth form massive, rounded or thickly encrusting. Colour orange rufous to brick red at the surface in dry specimens. Surface smooth, uneven, with irregularly meandering surface grooves. Consistency firmly spongy, resilient when wet; very hard, hardly compressible when dry. Ectosomal skeleton armed by tangential acanthostyles irregularly distributed. Choanosomal skeleton as a reticulate network with elliptical meshes (40–200 μm in diameter) of primary ascending spongin fibres and secondaries. Primary fibres (70–100 μm in diameter) cored by 4–8 acanthostyles, and echinated by the same acanthostyles. Secondary fibres (20–70 μm in diameter) not cored, in places echinated by acanthostyles. Megascleres of a single category. Acanthostyles 185–(226)–267 × 8–(12)–15 μm, straight to slightly curved, ornate by 15–23 whorls with eight spines each. **Habitat.** Shallow water, 20 m depth. **Geographic distribution.** Pacific Ocean. South Kuroshio, east Japan. Zamami Island (type locality), Ryukyu Archipelago, Japan. **Remarks.** Also recorded from Indonesia (de Voogd et al. 2008).

### *Agelas nemoechinata* Hoshino, 1985

Fig. 18

**Description** (modified from Hoshino 1985). Growth form massive or thickly encrusting.

Consistency spongy, resilient when wet, hard and difficult to compress when dry. Colour madder brown at the surface to rufous-orange in the interior when dry. Oscules from 1–3 to 5–8 mm in diameter. Ectosomal skeleton not reported. Choanosomal skeleton reticulate network with elliptical meshes (50–250 μm in diameter) of spongin fibres. Primary and secondary fibres almost indistinguishable (20–50 μm in diameter) and cored by 1–3 acanthostyles, occasionally not cored, only slightly echinated. Megascleres verticillate of a single category. Acanthostyles 170–(189)–210 × 9–(11)–13 μm, straight to gently curved, ornate by 16–23 regular whorls, each with eight spines. **Habitat.** Shallow water, 20 m depth. **Geographic distribution.** North Pacific Ocean. Recorded from south Kuroshio (east Japan). Type locality: Zamami Island, Ryukyu Archipelago. **Remarks.** Also recorded from Indonesia (de Voogd et al. 2008). These authors report acanthostyles occasionally sharply pointed at both ends (oxeas), as previously reported also in the original figures.

### *Agelas novaecaledoniae* Lévi & Lévi, 1983

Fig. 19

**Description** (modified from Lévi and Lévi 1983). Growth form as a thick blade (18 cm in height, 12.5 cm in diameter, 1–4 cm in thickness). Consistency elastic. Colour ochre brown. Surface irregularly cavernous and strongly hispid. Oscules (2–3 mm in diameter) numerous, 3–8 mm apart. Ectosomal skeleton armed by spicules. Choanosomal skeleton as a reticulate network of fibres (100 × 20 μm in diameter) laminar and fibrillar, rarely echinate at the sponge basal portion. Megascleres of two categories. Acanthostyles 100–190 × 5–8 μm (7–10 with spines) straight or slightly curved, verticillate by 12–17 whorls of spines. Acanthoxeas slightly curved (120–250 × 5 μm) verticillate by 12–18 whorls of spines. **Habitat.** Deep water, 390–395 m of depth. **Geographic distribution.** Pacific Ocean. Recorded from New Caledonia. **Remarks.** Known only from the type locality.

### *Agelas robusta* Pulitzer-Finali, 1982

Fig. 20

**Description** (modified from Pulitzer-Finali 1982). Growth form encrusting (5 mm in thickness) to massive, roundish (6 × 3 cm in diameter). Cavernous (clathrous) structure, with narrow channels through the entire sponge. Consistency tough and resilient. Colour dull orange, dull yellowish-brown. Ectosomal skeleton not reported. Choanosomal skeleton reticulate with irregular meshes of pale spongin fibres (38–80 μm in diameter) abundantly echinate by acanthostyles. Megascleres of a single category. Acanthostyles very stout, verticillate (170–250 × 14–30 μm spine included) with 11–12 whorls of short, blunt spines. **Habitat.** Not reported. **Geographic distribution.** Pacific Ocean. Recorded from Hong Kong, Southern China. **Remarks.** Known only from the type locality. At present for this species only three slides are available after damaging of type materials during the recent Genova flood in 2014 (October).

### *Agelas semiglabra* Pulitzer-Finali, 1996

Fig. 21

*Agelas semiglaber* Pulitzer-Finali, 1996

**Description** (modified from Pulitzer-Finali 1996). Growth form encrusting, very small. Skeleton architecture not reported. Megascleres of single category and two size classes. Acanthostyles type I verticillate (230–375 × 11–16 μm), spiny only towards the tips. Acanthostyles type II (75–100 × 3.5 μm) verticillate, entirely spiny. **Habitat.** Shallow water. **Geographic distribution.** Pacific Ocean. Recorded from Bismarck Sea, known only from Papua New Guinea (type locality). **Remarks.** The specific epithet ending with –*er* is masculine despite the gender *Agelas* is feminine, as a consequence the ending must be changed into –*ra.* Because of the overlap in spicule dimension de Voogd et al. (2008) report that is not possible to distinguish different size categories.

### *Agelas* spp.

Several findings from the Seychelles Archipelago (Thomas 1973) and Kenya (Pulitzer-Finali 1993). The revisitation of the 11 *Agelas* specimens slides of the Pulitzer-Finali collection from East Africa highlighted the presence of only styles in the spicular complement with notable variability of thickness, length, and spinosity. This confirms his opinion: ‘It would be inappropriate at the moment to try to identify some of them with established species and to propose new species.’ Also in this case the original material is not available after damaging of types during the recent Genova flood in 2014 (October).
